# Effects of Processing Parameters on the Structure and Mechanical Property of PVDF/BN Nanofiber Yarns

**DOI:** 10.3390/polym17141931

**Published:** 2025-07-13

**Authors:** Jincheng Gui, Xu Liu, Hao Dou

**Affiliations:** 1 School of Textile Science and Engineering, Xi’an Polytechnic University, Xi’an 710048, China; usersjc@foxmail.com (J.G.); liuuxuuu@outlook.com (X.L.); 2 Key Laboratory of Textile Industry for Silk Products in Medical and Health Use, Soochow University, Suzhou 215123, China

**Keywords:** electrospinning, PVDF, BN, nanofiber yarn, mechanical property

## Abstract

The increasing demand for light and comfort smart wearable devices has promoted the cross-integration of textile technology with nanomaterials and nanotechnology. As a potential candidate with excellent piezoelectricity, PVDF has been processed into different forms used for flexible sensors but shows limited practicality due to their discomfort and stiffness from non-yarn level. In this study, PVDF/BN nanofiber yarns (NFYs) were successfully fabricated via conjugated electrospinning. The effects of BN concentration, stretching temperature, and stretching ratio on the structural morphology and mechanical performance of the NFYs were systematically investigated. The results show that under the stretching temperature of 140 °C and stretching ratios of 3.5, smooth 1% PVDF/BN NFYs with highly oriented inner nanofibers can be successfully fabricated, and the breaking strength and elongation of composite NFYs reached 129.5 ± 8.1 MPa and 22.4 ± 3.8%, respectively, 667% higher than the breaking strength of pure PVDF nanoyarns. Hence, with the selection of appropriate nanofiller amounts and optimized post-treatment process, the structure and mechanical property of PVDF NFYs can be significantly improved, and this study provides an effective strategy to fabricate high-performance nanoyarns, which is favorable to potential applications in wearable electronic devices and flexible piezoelectric sensors.

## 1. Introduction

With the rapid development of modern material science and electronic technology, smart wearable textiles that integrate flexible electronic devices with traditional textile structures have attracted increasing attention [1,2,3,4]. These textiles offer excellent flexibility, high sensitivity, and multifunctionality, showing great promise in application areas such as health monitoring, human–computer interaction, electronic skin, soft robotics, and intelligent protection [5,6,7,8,9]. However, realizing smart textiles that are lightweight, highly flexible, stable, and comfortable remains a significant challenge.

Nanofibers with a diameter below 1 μm have a large specific surface area as well as high porosity and exhibit significant potential for various technical and commercial applications [10,11]. In recent years, electrospinning has been widely used in the preparation of nanofibers due to its simplicity, economy, and ease of assembly. But typically electrospun products are nonwoven membranes consisting of randomly oriented nanofibers, resulting in poor mechanical property and structural durability [12]. It is difficult for them to meet the wearable requirements of fabrics, and they are difficult to process into smart wearable textiles [12,13]. Yarns are an assembly of various fibers and play a crucial role in the formation of fabrics as they undergo weaving, knitting, and other manufacturing processes [14,15]. Hence, nanofiber yarns (NFYs) with enhanced mechanical and functional properties connect nanotechnology with textile engineering, creating new opportunities to address the increasing demand for flexible smart textiles [16,17].

Until now, conventional electrospinning has been modified in different ways to manufacture high quality NFYs, and various polymers have been successfully transferred into NFYs with different functions [18,19,20,21,22,23]. Electrospun PVDF nanofibers display excellent piezoelectricity and ferroelectricity and have been actively utilized in piezoelectric nanogenerators, sensors, and energy harvesting [24,25]. Furthermore, the addition of different nanofillers has demonstrated effective improvement on the polar β-phase contents of electrospun PVDF nanofibers and therefore increased the piezoelectricity for various sensing applications [26]. As an outstanding nanofiller, boron nitride (BN), which has been widely used in the field of highly composite materials and flexible electronic materials [27], can significantly enhance the thermal and mechanical properties of PVDF. Dang et al. [28] enhanced the mechanical properties of electrospun PVDF nanofibers by introducing BN, and the tensile strength was increased by 150% compared with pure PVDF membranes. Kim et al. [25] combined modified BN with PVDF to prepare electrospun PVDFNFs-OLABN composites, with tensile strength and Young’s modulus increasing by 343% and 823%, respectively. Nevertheless, few studies have focused on the PVDF/BN nanofiber yarns suitable for actual wear and innovative development in smart textiles.

In this study, PVDF/BN nanofiber yarns (NFYs) were fabricated by conjugated electrospinning, and the effect of BN concentrations on the structure and mechanical property of PVDF/BN NFYs was investigated. Moreover, post treatments including different stretching temperatures and stretching ratios were introduced to further improve the structure and mechanical property of PVDF/BN NFYs. This strategy provides an outline for designing and manufacturing high-performance PVDF NFYs for promising smart wearable devices.

## 2. Materials and Methods

### 2.1. Materials

All chemicals used in the experiments were of analytical grade. PVDF (HSV900, molecular weight ~1,000,000) was purchased from Arkema, Paris, France. Boron nitride (BN, 1 μm, purity > 99%) was purchased from Zhongye New Material Co., Ltd., Qingdao, China. *N*,*N*-dimethylformamide (DMF, analytical grade) was purchased from Aladdin Biochemical Technology Co., Ltd., Shanghai, China, and Acetone was purchased from Sinopharm Chemical Reagent Co., Ltd., Shanghai, China.

### 2.2. Fabrication of Electrospun NFYs

Next, 10 wt% polymer solution was prepared by dissolving a certain amount of PVDF powder in the mixture of DMF and acetone at a volume ratio of 7:3 (*v*/*v*). The solution above was then stirred for 2 h at 70 °C to ensure that PVDF powder was fully dissolved to form a uniform and stable spinning dope. Finally, an ultrasonic cleaner was used for 15 min to remove air bubbles in the solution. In order to investigate the effect of BN contents on the electrospun PVDF/BN NFY, BN powders were added in the above PVDF solution to obtain PVDF/BN composite solutions with BN mass ratios of 1%, 3%, and 6%, respectively.

**Figure 1:**
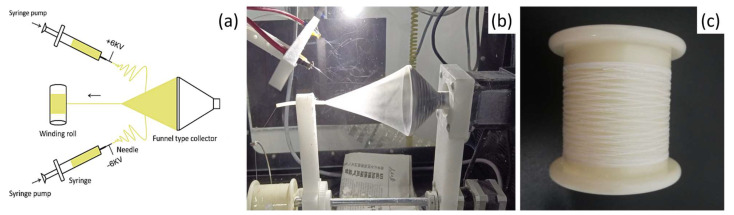
(**a**) Schematic diagram of conjugated electrospinning setup. (**b**) Photograph of conjugated electrospinning device. (**c**) Photograph of continuous as-spun PVDF/BN NFYs.

The electrospun nanofiber yarns (NFYs) were fabricated using a conjugated electrospinning technique, as shown in Figure 1a. This setup, based on previously reported designs [13,29,30], consists of two oppositely positioned steel needles, a rotating funnel-type collector, a winding roll, a guiding device, and two DC power supplies. The operation process is as follows: two nozzles separately connected to a DC power supply at +6 kV and −6 kV are arranged symmetrically with a spacing of 17 cm, and the solution was loaded into two syringes with metal needles. The flow rate for both nozzles was set as 0.6 mL/h. Nanofibers ejected from two nozzles were initially collected on the edge of the rotating funnel-type collector. A cone-shaped nanofiber thin film was immediately generated along the collector and twisted by the rotation of the collector at 200 rpm and then was drawn through the guiding device at 6 cm/min and finally wound onto the winding roll to form the continuous NFY shown in Figure 1b. The spinning experiment was carried out continuously, and the obtained as-spun NFY samples were shown in Figure 1c. In order to study the influence of post treatments on NFYs, 1% PVDF/BN NFYs were stretched at a stretch ratio (SR) of 2 (defined as the ratio of the final length to the original length of NFYs) at temperatures of 60 °C, 100 °C, and 140 °C, and further different stretch ratios (SR) determined by the length ratio of the stretched NFY and the as-spun NFY ranging from 2 to 3.5 at 140 °C were investigated. Finally, all stretched NFYs were further annealed at 150 °C under tension for one hour.

### 2.3. Characterization

Scanning electron microscopy (SEM, Quanta-450-FEG+X-MAX50, Eindhoven, The Netherlands) was used to observe the morphology of NFYs and inner nanofibers, which were sputter-coated for 90 s with gold layer 20–30 nm thick before observation. Several sections of NFYs and tens of nanofibers were randomly selected for each sample, and the diameter of NFYs and inner nanofibers was measured using Image J 1.45 software, respectively. Specifically, for each sample, at least 10 independent diameter measurements at randomly selected positions along the nanofibers were performed. The reported diameter values, precise to 1 nm, reflect statistical means and standard deviations calculated directly by Image J 1.45 software, indicating measurement variability rather than actual measurement precision. Such detailed statistical analysis ensures that the observed trends in morphology and structure are not accidental but reliably reflect the impact of varying BN concentrations and processing conditions. The orientation angle, representing the average degree of nanofiber alignment along the NFY axis, was manually measured using Image J 1.45 software by calculating the angle between individual nanofibers and the yarn axis in high-magnification SEM images. At least 15 fibers were measured per group to ensure statistical representativeness.

The tensile tests of all samples cut into 50 mm (length) were carried out by a universal testing device (UTM5205X, Shenzhen, China) with a clamping length of 10 mm at a stretching rate of 5 mm/min after all samples had been placed in a room with a constant temperature of 24 °C and humidity of 65% for 24 h. A pre-tension of 0.5 cN (centinewton) was applied to same straighten and properly position each yarn sample before starting the tensile test. The final results were the average value of at least 10 measurements for each sample, the tensile stress was calculated as the ratio of the breaking force to the cross section of the yarns, and the strain was recorded as the ratio of the elongated length to the gauge size based on the following Equations (1) and (2).

(1) $$\mathrm{tensile}\;\mathrm{stress}\;(\mathrm{MPa})=\frac{4\times \mathrm{breaking}\;\mathrm{force}\;(N)}{\pi \times \mathrm{average}\;\mathrm{diameter}\;\mathrm{of}\;\mathrm{yarn}\;{\left(\mathrm{mm}\right)}^{2}}$$

(2) $$\mathrm{strain}\;(\%)=\frac{\mathrm{elongated}\;\mathrm{length}\;(\mathrm{mm})}{\mathrm{gauge}\;\mathrm{size}\;(\mathrm{mm})}\times 100\%$$

## 3. Results and Discussion

### 3.1. Effect of BN Concentrations on the Structure of PVDF/BN NFYs

**Figure 2:**
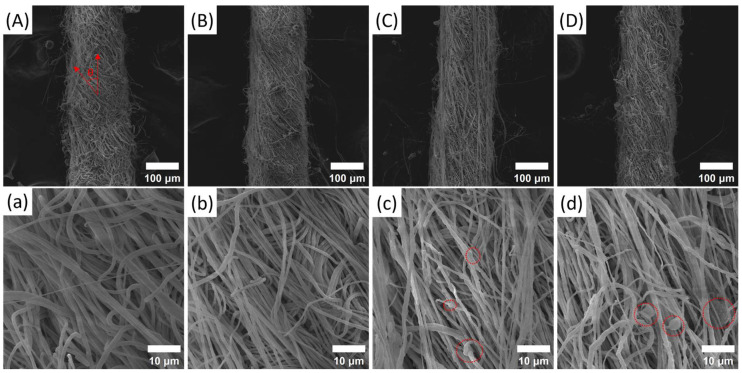
SEM images of PVDF/BN NFYs (**A**) 0 wt%, (**B**) 1 wt%, (**C**) 3 wt%, and (**D**) 6 wt% and of corresponding inner nanofibers (**a**) 0 wt%, (**b**) 1 wt%, (**c**) 3 wt%, and (**d**) 6 wt% with different BN concentrations. (**A**) shows the orientation angle measurement method used in characterization. The agglomeration mentioned in the article is marked in (**c**,**d**).

**Figure 3:**
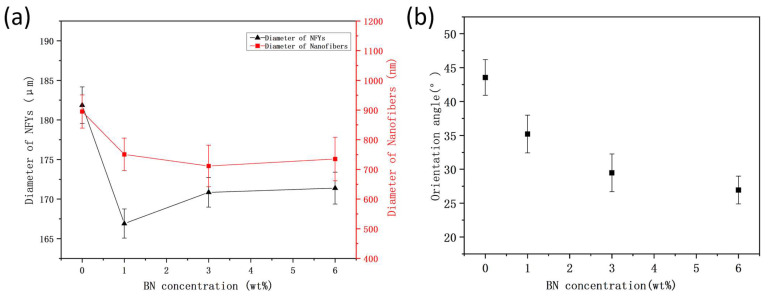
(**a**) Average diameters of PVDF/BN NFYs and inner nanofibers; and (**b**) orientation angle of nanofibers along NFYs axis at different BN concentrations.

In order to systematically explore the effect of BN concentrations on the morphology of PVDF NFYs, the surface morphology of NFYs is presented in Figure 2. The comparison results of the average diameter of the NFYs and their inner nanofibers are separately shown in Figure 3a.

As shown in Figure 2A–D, all NFYs consist of a large number of fine nanofibers and exhibit a bundled yarn structure with slight surface hairiness, which results from nanofibers being deposited preferentially in the triangular region of the funnel-type collector during conjugated electrospinning. The overall appearance of the NFYs remains consistent across different BN concentrations [27]. Based on the average of all samples, the NFY diameter is approximately 172 μm. However, as shown in Figure 3a, the 1% BN sample exhibits a notably smaller diameter (~166 μm), while the 0%, 3%, and 6% BN samples are closer to the mean value. In terms of fiber morphology, the nanofibers in the 0% and 1% BN samples shown in Figure 2a,b are smooth and uniform, whereas those in the 3% and 6% BN samples shown in Figure 2c,d become rougher and more irregular due to the likely agglomeration or uneven distribution of BN fillers, which may weaken the cohesion between nanofibers. This phenomenon was also observed by Zhang and was called the hot string formed by the modified BN (m-BN) decorated fibrous film surface [28]. In addition, the average diameter of inner fibers showed a decreasing trend from 895 ± 57 nm (0% BN) to 711 ± 70 nm (3% BN), followed by a slight increase to 735 ± 74 nm (6% BN), as shown in Figure 3a.

However, the standard deviations across samples (±57–73 nm) are relatively large and partially overlapping, suggesting that the observed diameter changes are within the range of measurement variation. This indicates that BN addition has only a limited effect on fiber diameter distribution under the current processing conditions and may be influenced by natural variability in solution viscosity and conductivity [16,31,32].

From the aspect of the orientation angles of nanofibers along NFYs axis in Figure 3b, it can be seen that with the increase in BN concentration, the orientation angle gradually decreases from 43.5 ± 2.6° to 26.9 ± 2.0°; this is because nanofibers with the BN addition are easier to stretch and manipulate during collection and are more likely to be arranged axially. On the other hand, although NFYs are weakly twisted when the funnel-type collector is slowly rotated, the nanofibers collected are still in a state of random arrangement like the products obtained from traditional electrospinning. So post treatments are considered to carry out.

### 3.2. Effect of BN Concentrations on the Mechanical Property of PVDF/BN NFYs

**Figure 4:**
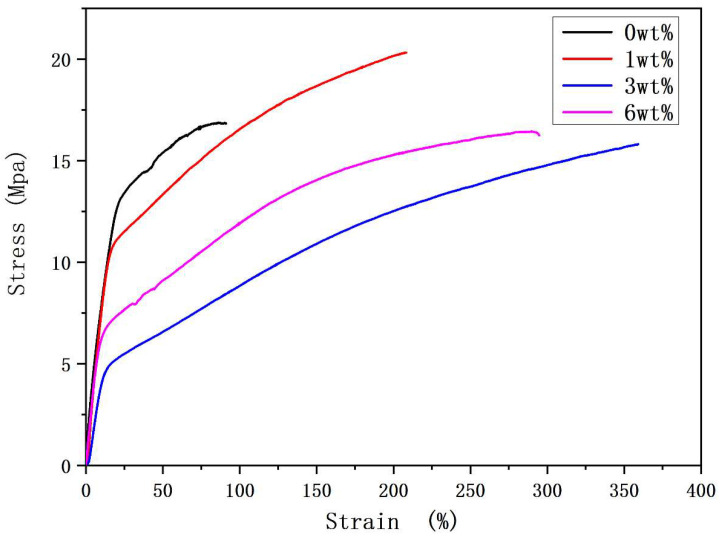
Stress-strain curves of PVDF/BN NFYs at different BN concentrations.

Figure 4 shows the stress-strain curve of PVDF-based NFYs at different BN concentrations. With the BN concentration increase from 0 to 1%, both the tensile strength and the breaking elongation of PVDF/BN NFYs clearly improve, from 16.9 ± 1.2 MPa to 20.4 ± 1.5 MPa [a 20.7% increase] and from 91.3 ± 6.6% to 208.2 ± 11.4% [a 128.0% increase], respectively, reaching the maximum. This could be due to be the uniform dispersion of BN fillers in the nanofibrils to form a certain “bridging effect” [33]. However, when the BN content is further increased to 3 wt% and 6 wt%, the breaking stress of PVDF/BN NFYs shows a downward trend, but the breaking elongation keeps rising. This trend agrees well with results of the tensile strength of PVDF/m-BN or PVDF-OLABN nanofibrous films [25,28]. On the one hand, the decrease in orientation angles illustrated in Figure 4 leads to weak cohesion and friction between nanofibers; on the other hand, irregular nanofibers with excessive BN particles seen in Figure 2c,d are easy to slip, resulting in the poor-quality reducing strength of NFYs.

Consequently, the introduction of a proper BN amount can significantly improve the structural uniformity, orientation, and mechanical property of PVDF NFYs, but excessive addition may cause uneven diameter of nanofibers and poor quality of NFYs; thus, 1% PVDF/BN will be selected for post-treatment candidate. While higher BN contents such as 3% or 6% might offer marginal gains in elongation, the adverse effects on stress and structural uniformity outweigh the benefits. Hence, 1% BN presents the optimal trade-off between reinforcement and processability.

### 3.3. Effect of Stretching Temperatures on the Structure of 1% PVDF/BN NFYs

In order to systematically study the effect of temperature on 1% PVDF/BN NFYs, the stretching temperature was set above the glass transition temperature (Tg = −35 °C) and below the melting point (Tm = 165 °C) of PVDF, to facilitate molecular chain rearrangement while avoiding thermal degradation [34]. Temperature conditions of 60 °C, 100 °C, and 140 °C were selected to represent low, intermediate, and high thermal activation levels, respectively, for a comparative evaluation of structural and mechanical transformations under thermal drawing.

**Figure 5:**
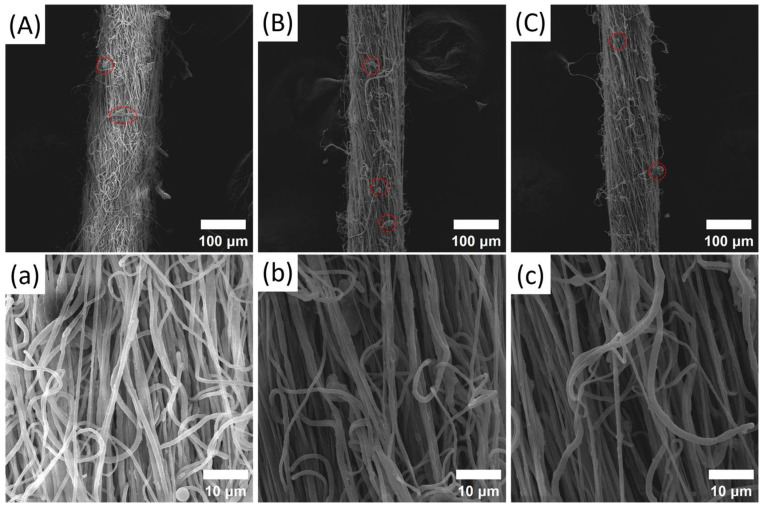
SEM images of 1% PVDF/BN NFYs (**A**) 60 °C, (**B**) 100 °C, and (**C**) 140 °C and of corresponding inner nanofibers (**a**) 60 °C, (**b**) 100 °C, and (**c**) 140 °C at different stretching temperatures. The bead-like defects mentioned in the article are marked in (**A**–**C**).

**Figure 6:**
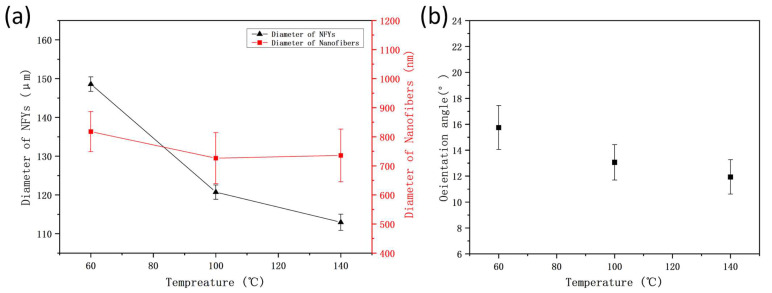
(**a**) Average diameters of 1% PVDF/BN NFYs; and (**b**) orientation angle of nanofibers along NFYs axis at different stretching temperatures.

Figure 5A–C, a–c present the SEM images of 1% PVDF/BN NFYs and corresponding inner nanofibers at different stretching temperatures, respectively. It is evident that all NFYs like bundles are composited of densely aggregated nanofibers aligned along the yarn axis, showing improved orientation. At the surface of the NFYs, a small number of disordered nanofibers with bead-like defects were observed (highlighted in Figure 5), especially under higher temperature conditions. The appearance and evenness of NFYs seems not to be affected by hot stretching, but the average diameter of 1% PVDF/BN NFYs gradually decreases from 148.6 ± 1.9 μm at 60 °C to 112.9 ± 2.1 μm at 140 °C according to Figure 6a, indicating that the molecular chains are more likely to be oriented under the thermal motion of the polymer chain segments at high temperature. For the average diameter (~760 nm) of inner nanofibers at different temperatures, no obvious difference is observed according to Figure 6a.

In addition, as shown in Figure 6b, with the increase in the heat treatment temperature, the orientation angle of the nanofibers inside the NFYs along the axial direction shows a gradual decrease from 15.7 ± 1.7° at 60 °C to 11.9 ± 1.3° at 140 °C. These values are all significantly lower than those of as-spun PVDF/BN NFYs without any thermal treatment, whose orientation angles remain above 26.9 ± 2.0° in all compositions, as shown in Figure 3b. This indicates that heat treatment plays a key role in promoting molecular chain alignment and fiber orientation, regardless of BN content. Although both NFY evenness and fineness are optimized, the nanofibers in the relaxed state are still not capable of fully straightening due to the limited stretching ratio, as observed in Figure 5a–c.

### 3.4. Effect of Stretching Temperatures on the Mechanical Property of 1% PVDF/BN NFYs

**Figure 7:**
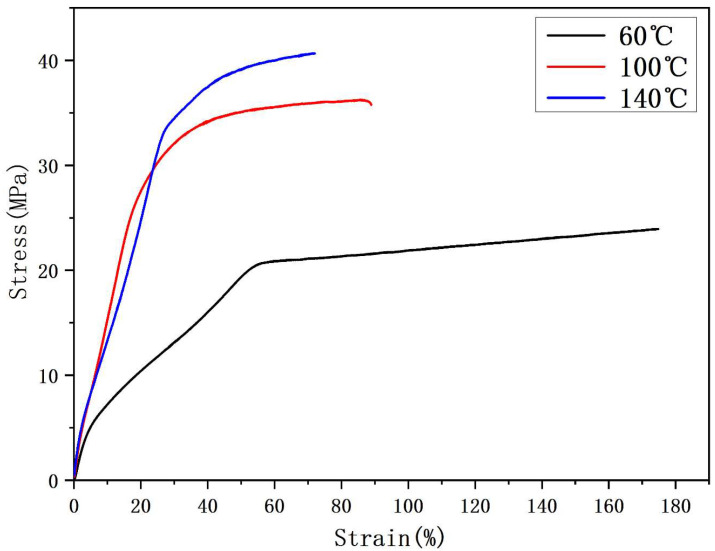
Stress-strain curves of 1% PVDF/BN NFYs at different stretching temperatures.

To analyze the effect of stretching temperatures on the mechanical properties of 1% PVDF/BN NFYs, the stress and strain behavior was evaluated based on Figure 7. The results show that the mechanical properties of 1% PVDF/BN NFYs are highly sensitive to thermal stretching conditions. Specifically, the breaking tensile strength increased from 24.3 ± 1.7 MPa at 60 °C to 42.8 ± 2.8 MPa at 140 °C. Compared to the untreated 1% PVDF/BN NFYs (20.4 ± 1.5 MPa, see Figure 4), this represents a 109.8% improvement. Moreover, this value is also 153.3% higher than that of pure PVDF NFYs (16.9 ± 1.2 MPa), confirming the synergistic effect of BN reinforcement and thermal drawing.

At the same time, the elongation at break decreased with increasing temperature, from 174.8 ± 9.3% at 60 °C to 72.1 ± 5.9% at 140 °C. This phenomenon can be attributed to the enhanced crystallinity and molecular chain alignment of PVDF/BN NFYs under thermal stretching [2,7], as well as increased inter-fiber interactions and restricted slippage of nanofibers at elevated temperatures [12].

In summary, increasing the stretching temperature facilitates better alignment of nanofibers and the formation of a compact structure. These improvements contribute to significantly enhanced mechanical properties in 1% PVDF/BN NFYs.

### 3.5. Effect of Stretching Ratios on the Structure of 1% PVDF/BN NFYs

In order to systematically study the effects of different stretching ratios (SR) on the structure and morphology of PVDF/BN nanofiber yarns, 1% PVDF/BN NFYs were subjected to hot stretching with SRs of 2, 2.5, 3, and 3.5 at 140 °C.

**Figure 8:**
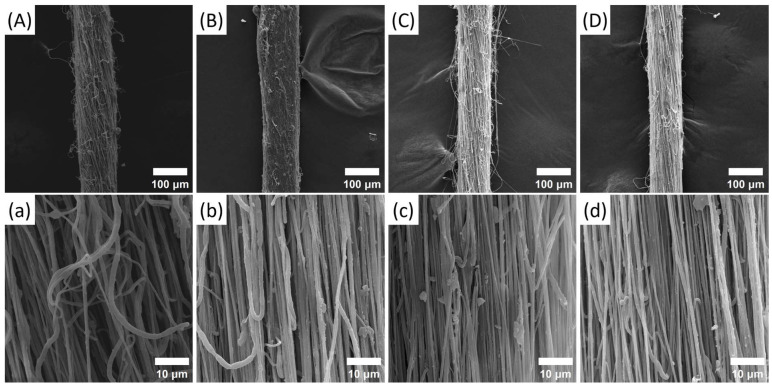
SEM images of 1% PVDF/BN NFYs (**A**) 2 SR, (**B**) 2.5 SR, (**C**) 3 SR, and (**D**) 3.5 SR and of corresponding inner nanofibers (**a**) 2 SR, (**b**) 2.5 SR, (**c**) 3 SR, and (**d**) 3.5 SR at different stretching ratios. Note: Figure 8a is identical to Figure 5c, as both represent the same sample condition of 1% PVDF/BN NFYs treated at 140 °C under a stretching ratio of 2 SR. The image is repeated here for clarity in the context of stretching ratio analysis.

The results in Figure 8 indicate that NFYs are increasingly acting as a whole, and the curly nanofibers gradually become straight and present a highly oriented arrangement with the increase in the stretching ratio, which leads to the hairiness reduction on NFYs’ surface and the compactness promotion between nanofibers. Thereby, the uniformity and evenness of NFYs can be improved by applying hot stretching as much as possible.

**Figure 9:**
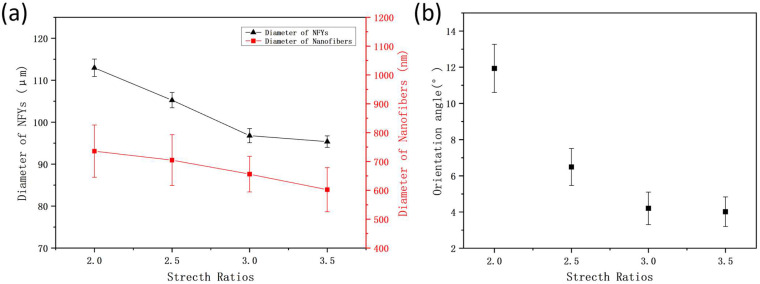
(**a**) Average diameters of 1% PVDF/BN NFYs; and (**b**) orientation angle of nanofibers along NFYs axis at different stretching ratios.

At the same time, the average diameters of 1% PVDF/BN NFYs and their corresponding inner nanofibers, as shown in Figure 9a, show a general decreasing trend from 112.9 ± 2.1 μm at 2 SR to 95.4 ± 1.4 μm at 3.5 SR, and from 736 ± 91 nm to 602 ± 77 nm, respectively. Although the inner fiber diameters fall within overlapping error ranges, the overall tendency suggests radial attenuation and compaction during hot stretching, likely due to reduced interfiber spacing and molecular chain realignment. Furthermore, the evolution of fiber orientation was confirmed by Figure 9b. With increasing stretch ratio, the orientation angle of the nanofibers along the NFY axis decreases sharply before 3 SR and more gradually thereafter. Notably, near-parallel alignment is observed at 3.5 SR, with an orientation angle of only 4.0 ± 0.8°, indicating a completed structural transformation from coiled to straight configuration.

### 3.6. Effects of Stretching Ratios on the Mechanical Property of 1% PVDF/BN NFYs

**Figure 10:**
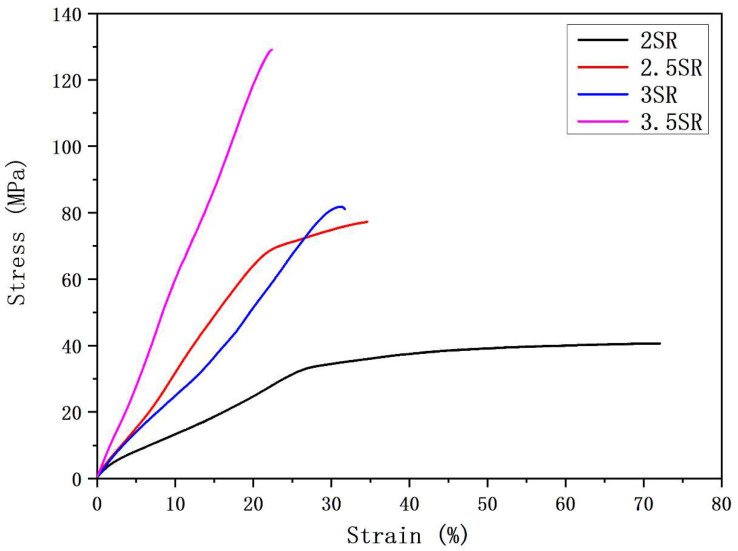
Stress-strain curves of 1% PVDF/BN NFYs at different stretching ratios.

It can be observed from Figure 10 that the breaking stress of 1% PVDF/BN NFYs significantly increases from 42.8 ± 5.4 MPa to 129.5 ± 8.1 MPa, while the elongation at break shows a downward trend from 72.1 ± 9.6% to 22.4 ± 3.8%. Compared with the same composite without any stretching treatment (20.4 ± 1.5 MPa, see Figure 4), this result indicates that post treatment has a crucial effect on improving the mechanical property of NFYs. Moreover, when the stretching ratio is 3.5, the stress of 1% PVDF/BN NFYs is about 667% higher than that of the untreated pure PVDF NFYs (16.9 ± 1.2 MPa), which further reflects the synergistic effect of BN reinforcement and molecular alignment under thermal drawing.

Two reasons can mainly account for the effect of hot stretching on the mechanical property of NFYs. On the one hand, the strength of NFYs is proved to be directly proportional to its diameter based on Peirce’s Theory, which means the smaller the yarn, the lower the probability of weak links [12]. On the other hand, both the crystallinity of NFYs and molecular orientation of nanofibers rise through the reorientation of loose or curled molecular chains inside the nanofibers along the axial direction upon stretching force, which is applied at high temperature [7,12,35]. Besides, the uniform dispersion of BN within PVDF nanofibers and the interfacial interaction between PVDF and BN facilitate effective load transfer [27,32].

## 4. Conclusions

In conclusion, high-strength PVDF NFYs with BN used as reinforced fillers were successfully fabricated by a combination of conjugated electrospinning and post treatment, and the effects of different BN concentrations, stretching temperatures, and stretching ratios on the structure and mechanical property of PVDF/BN NFYs were systematically investigated. Among all the investigated factors, BN concentration influenced fiber uniformity and diameter; stretching temperature modulated nanofiber alignment and packing; and the stretching ratio had the most pronounced effect on mechanical strength due to its direct control over fiber orientation and crystallinity. The results show that smooth and uniform PVDF/BN NFYs with little hairiness can be successfully fabricated by selecting appropriate BN concentration and stretching temperature and ratios, leading to an obvious decrease in average diameter of both NFYs (from 181.9 ± 2.3 μm to 95.4 ± 1.4 μm) and inner nanofibers (from 895 ± 56 nm to 602 ± 77 nm) and a corresponding enhancement in the stress of treated 1% PVDF/BN NFYs with 129.5 ± 8.1 MPa as compared with pure PVDF NFYs with 16.9 ± 1.2 MPa.

Overall, a proper BN amount can have a critical influence on the structural uniformity, orientation, and mechanical property of PVDF NFYs due to the interaction between PVDF and BN as well as the property change of electrospinning solutions. In addition, the reasonable selection of processing parameters like stretching temperatures and stretching ratios provides an effective way to contribute to the high integrity of NFYs and greatly improves the mechanical performance, which is beneficial to wearable electronic products such as advanced functional textiles.

## Data Availability

The original contributions presented in this study are included in the article. Further inquiries can be directed to the corresponding author.
